# A Hyper-Elastic Creep Approach and Characterization Analysis for Rubber Vibration Systems

**DOI:** 10.3390/polym11060988

**Published:** 2019-06-04

**Authors:** Dingxin Leng, Kai Xu, Liping Qin, Yong Ma, Guijie Liu

**Affiliations:** 1 Department of Mechanical and Electrical Engineering, Ocean University of China, Qingdao 266024, China; lengdingxin@126.com (D.L.); user_ xukai@126.com (K.X.); 2 Seventh thirteen Institute of China Shipbuilding Industry Corporation, Zhengzhou 450009, China; qxjt008@163.com (L.Q.); myourever@sohu.com (Y.M.)

**Keywords:** finite element analysis, creep behavior, rubber, vibration system, hyper-elasticity, creep damage

## Abstract

Rubber materials are extensively utilized for vibration mitigation. Creep is one of the most important physical properties in rubber engineering applications, which may induce failure issues. The purpose of this paper is to provide an engineering approach to evaluate creep performance of rubber systems. Using a combination of hyper-elastic strain energy potential and time-dependent creep damage function, new creep constitutive models were developed. Three different time-decay creep functions were provided and compared. The developed constitutive model was incorporated with finite element analysis by user subroutine and its engineering potential for predicting the creep response of rubber vibration devices was validated. Quasi-static and creep experiments were conducted to verify numerical solutions. The time-dependent, temperature-related, and loading-induced creep behaviors (e.g., stress distribution, creep rate, and creep degree) were explored. Additionally, the time–temperature superposition principle was shown. The present work may enlighten the understanding of the creep mechanism of rubbers and provide a theoretical basis for engineering applications.

## 1. Introduction

Vibration mitigation is an essential design requirement in several industries, such as aerospace, rocket-engine, and automotive [1]. Passive damping technology often utilizes viscoelastic materials to decrease the vibratory level transmitted and the vibration field generated. Traditional viscoelastic materials are rubbers, which are widely utilized for years of service [2]. In practice, when a constant load is applied to a rubber material, its deformation is not a constant; it gradually increases with time, which is known as creep. The creep presents a time-dependent characteristic, which induces the dimensional instability of rubber products over their expected lifetime and may finally lead to early failure and deteriorate the vibration mitigation performance. Hence, it is important to accurately predict the creep behaviors of rubbers so that the fracture failure due to the creep effect can be prevented.

**Figure 1:**
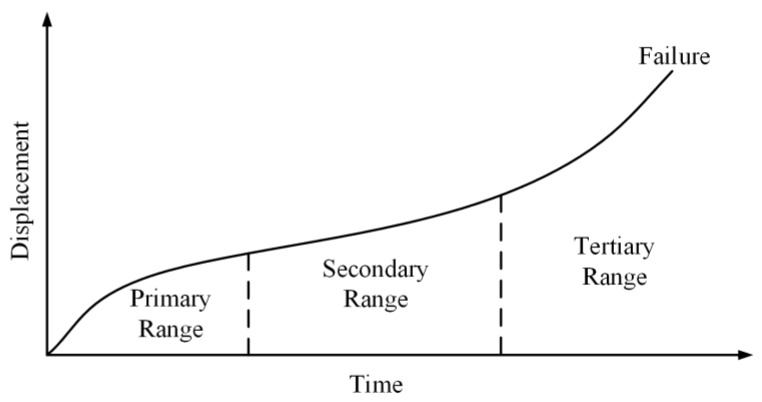
Strain as a function of time for the three creep stages [3].

Creep is typically classified in three stages, as shown in Figure 1 [3]. The first one is primary creep or transient creep, which is related to the physical rearrangement of polymer chains (e.g., bond stretching/bending and crosslinking between chains of rubber materials) [4]. In the primary creep stage, the strain rate is initially high and reduces with time. When the strain rate diminishes to a minimum value, the secondary stage begins and an obvious time-dependent behavior is presented, that is, the stain increases remarkably after an important length of elapsing time. The third stage is termed as the creep failure stage, in which the creep resistance is weakened and fracture inside the rubber is presented. In engineering applications, creep analysis practically considers the first two creep stages.

The reliability of creep predictions is dependent on the application of computational models. Hyper-elastic models are commonly utilized to describe the nonlinear properties of rubber materials and rubber-based devices. In mathematical expression, the hyper-elasticity of rubber is issued from the strain energy density, which is a function of principal invariants related to the Cauchy–Green deformation tensors and the Jacobian matrix of the deformation gradient [5]. Widely applied hyper-elastic models include the Mooney–Rivlin model [6,7], the Biderman model [8], the Ogden model [9], the Yeoh model [10], the Neo–Hookean model [5], and polynomial series [11]. The parameters in these phenomenological models are identified according to the experimental data. Additionally, some hyper-elastic models are based on microscopic responses of polymer chains in the network of rubber materials, e.g., six or eight constrained-chain models [12,13]. Hyper-elastic models focus on portraying nonlinear force–deflection responses of rubbers; however, they cannot describe the time-dependent creep responses due to the models without referring to the elapsed loading time [14].

Creep behavior is attributed to the time-related viscoelasticity of rubber materials. The description of viscoelastic behavior can be achieved by taking into account the appropriate amounts of elastic and damping elements into viscoelastic models. Typical computational models, such as the Maxwell element and the Kelvin–Voight model, are suitable for depicting linearly viscoelastic properties; however, long-term nonlinear creep responses are not accurately predicted. Subsequently, some complex viscoelastic models were proposed. Skrzypek [15] proposed a creep model by modifying Boltzmann’s superposition principle to describe nonlinear creep laws, in which time-dependent creep strain was studied. Lee [16] established a modified viscoelastic model in prony series to study creep characteristics of compressed rubber products and a finite element analysis was presented. Majda [3] developed a modified Burger model with tunable damping and stiffness coefficients for calculating creep deformation, which was validated by experimental results. Although these complex viscoelastic models can forecast creep nonlinearity, their complexity results in time-consuming calculations and substantial creep experimental results must be inputted to determine large numbers of model parameters, which limits the practical application.

It is noteworthy that a mechanical model for creep analysis with high efficiency and reasonable accuracy is particularly attractive. Recently, Luo proposed an easily implemented creep damage model for predicting long-term creep characteristics of polyisoprene rubbers [17]. However, his work focused on the creep analysis under a fixed loading level and temperature and the validation for various conditions was not considered. In practice, rubber materials are commonly used in various conditions (loading level, temperature, humidity, oxygen aging) and some studies revealed that the loading level and ambient temperatures are major factors which largely affect the creep characteristics [3]. It is also emphasized that creep performances of rubbers under different loading conditions and temperatures are significant for engineering designers [18]. Some related work has been conducted, e.g., Rivin [18] carried out creep tests of compressed rubber components under different levels of static loading and the correspondence characteristics were discussed. Oman [19] observed the influence of test programs and loading conditions on the creep responses of rubbers and different creep performances were compared. Wang [20] performed a laboratory evaluation on the creep viscosity and stiffness of tire rubber under low and high temperatures and the temperature-related creep stability was analyzed. These studies are mainly experimental works, in which creep deformation is qualitatively discussed from the test results. Nevertheless, due to observation difficulties, the stress variation, creep rate, and creep degree are not fully addressed, but such information is beneficial to explore creep mechanism for the engineering design of rubber systems.

As mentioned above, although creep behaviors of rubbers have been theoretically studied for decades, it has been solved by the viscoelastic mechanical models. However, the viscoelastic model is not an optimal solution for engineering applications. Different from the previous work, the present study investigates creep performance with a modified hyper-elastic mechanical model with a time-dependent creep damage function. The integration of the proposed model in the commercial finite element software Abaqus is provided by utilizing user subroutines. Its validation is presented by experimental work. By utilizing the proposed model for predicting creep behaviors of rubber vibration systems, the engineering potential of the proposed model is validated. The detailed arrangement of the present work is as follows. The experimental testing of rubber materials is shown in Section 2. The constitutive equation and its numerical implementation in the finite element software are provided in Section 3. The identified parameters of the proposed model are determined by the experimental data. Section 4 validates the developed approach and discusses the creep performances of rubber materials under multi-level loadings and temperatures. For further evaluating engineering potential, the proposed approach is utilized for predicting creep performances of rubber vibration systems. The main conclusions are drawn in Section 5.

## 2. Experimental Testing

### 2.1. Quasi-Static Compression Tests

**Figure 2:**
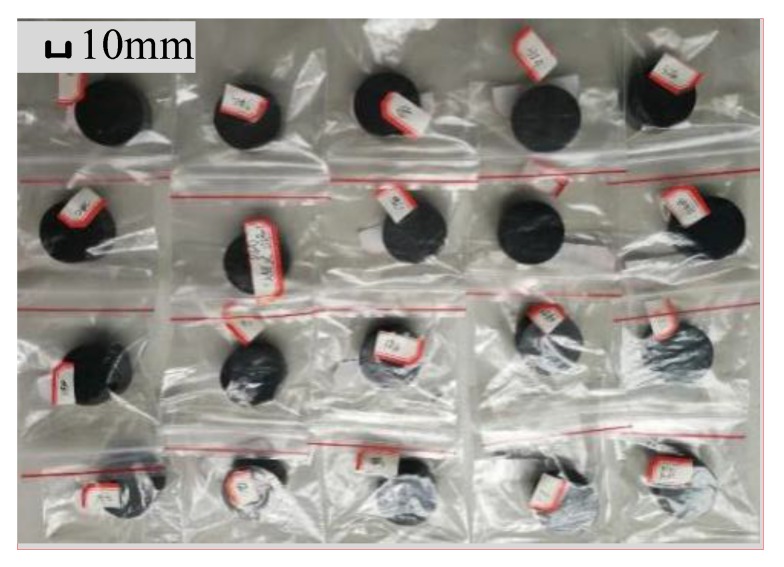
Rubber samples for quasi-static compression loading.

**Figure 3:**
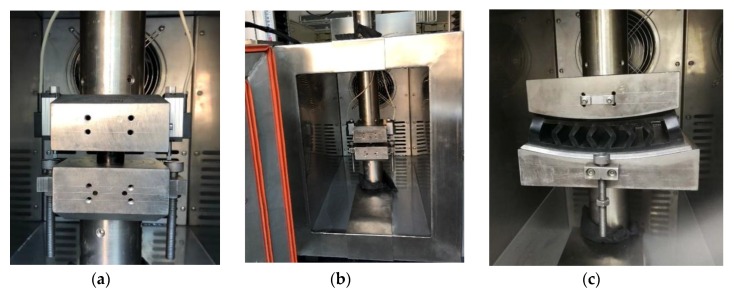
Experimental setup: (**a**) Quasi-static compression of rubber sample, (**b**) creep compression of rubber sample, and (**c**) creep compression of rubber device.

A quasi-static compression test was performed before the creep experiment to analyze nonlinear hyper-elasticity of the rubber materials. Acrylonitrile-butadiene rubbers (Yi-Ke Rubber Manufacturing Corporation, Qingdao, China) were utilized for preparing the rubber samples. The measurements of the studied cylinder-shaped samples were 29.0 mm in diameter and 12.5 mm in thickness, as shown in Figure 2. The loading was applied along the axial direction of the sample. All the tests were performed using a servo-hydraulic testing system (type: WDW-50) in which the load cells with ±1% accuracy and the displacement sensor with ±0.01 mm accuracy were equipped. The frequency range of this machine was 0–20 Hz. The rubber sample was constrained between two plates in the experimental system. The experimental setup is shown in Figure 3.

**Figure 4:**
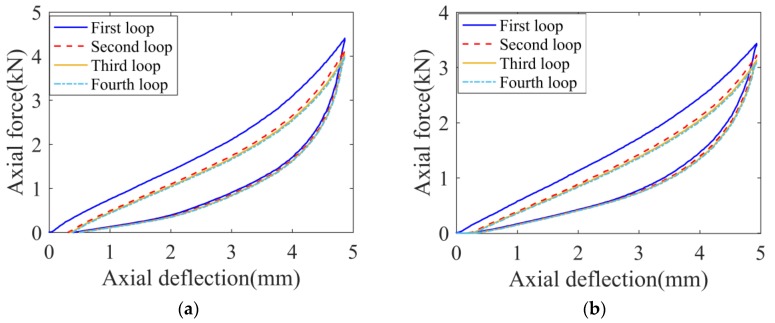
Experimental compression force-deflection history for the rubber sample. (**a**) 23 °C and (**b**) 55 °C.

In the load-deflection compression tests, the specimens were compressed at 25 mm/min according to the standard test method [21]. The compression was applied without interruption up to 39% relative deflection for axial compression. Four loading–unloading cycles were performed in tested specimens. The temperatures for the two tests were maintained at 23 ± 1 °C and 55 ± 1 °C, respectively and dry air with relative humidity was less than 30%. The load-deflection responses of the rubber samples in the multiple loading and unloading cycles are shown in Figure 4.

Figure 4 shows a representation of the Mullins effect [22], and a permanent set is presented upon unloading which denotes viscoelastic effects such as hysteresis. Due to the Mullins effect, the “loading softening” of rubber samples is clearly indicated, especially in the first and second loops, i.e., the force value from the second loading is lower than that from the first loading at a given deformation, approximately 16.7%. The stable force-deformation loop is presented in the third cycle. Hence, in the following creep tests, before experimental data was recorded, several loading–unloading cycles were conducted to diminish the Mullins effect. Additionally, compared with Figure 4a,b, with the temperature increasing, the slope of the loading curve of the rubber sample reduces, which indicates that the equivalent stiffness of rubber is weakened.

### 2.2. Creep Compression Tests

Creep tests of rubber materials under multiple levels of loading and temperature were conducted. The sample had a diameter of 12.5 mm and a height of 29.0 mm and three levels of applied loading (e.g., 1.5 kN, 2.0 kN, and 2.5 kN) and two levels of temperature (e.g., 23 ± 1 °C and 55 ± 1 °C) were assumed along the vertical direction. All the creep tests lasted 48 h. The measurement of deformation was performed using a non-contact laser extensor displacement sensor operated at the measurement accuracy of 0.1%. The creep experimental setup is shown in Figure 3b.

**Figure 5:**
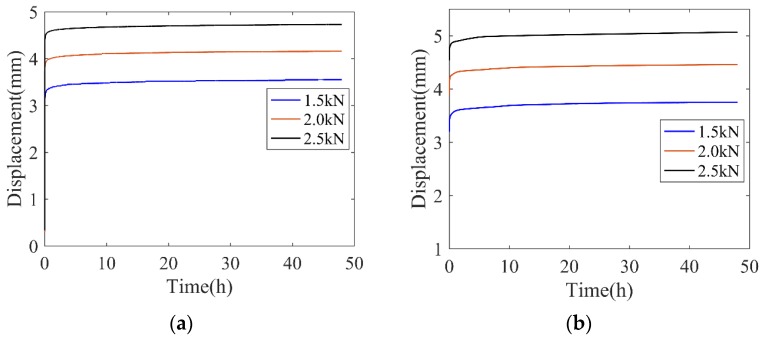
Experimental creep history for rubber materials. (**a**) 23 °C and (**b**) 55 °C.

The time-deformation curves of rubbers during the creep tests are shown in Figure 5.

In Figure 5, it is shown that, in the initial creep stage, the deformation largely increases, which is assumed to be accelerated creep. In the long-term creep stage, the deformation gently increases with elapsed time, which is termed as stable creep. Compared with Figure 5a,b, the creep deformation increases with the increase in temperature and at a fixed temperature, the slope in the long-term creep stage approximately keeps a constant in different levels of applied loadings, which demonstrates that the creep characterization is less sensitive to loading levels than temperature.

**Figure 6:**
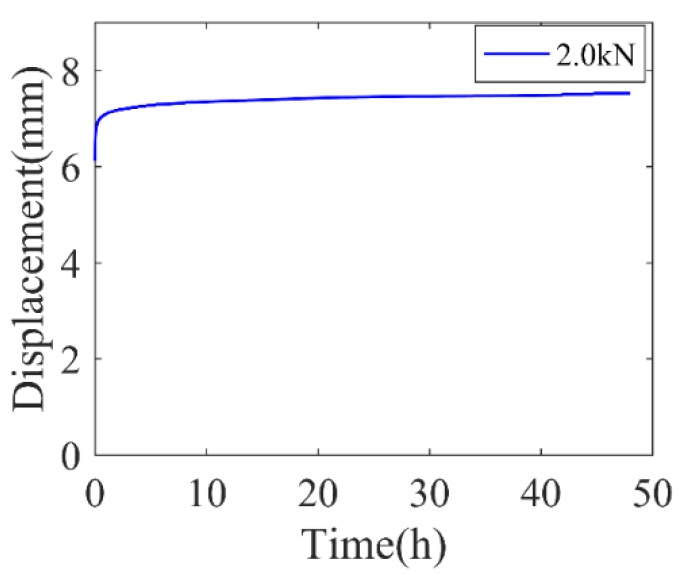
Time-deformation curve of rubber vibration system in creep test.

Additionally, creep tests of the rubber vibration system were conducted. At room temperature (e.g., 23 ± 1 °C), 2.0 kN loading was applied along the normal direction for 48 h. The test setup was shown in Figure 3c. The time-deformation curve in creep test at 23 °C is presented in Figure 6.

## 3. Numerical Simulation

### 3.1. Constitutive Model

In the analysis of experimental data, the rubber materials in creep tests showed nonlinear elasticity in the initial load/deflection characteristics and time-dependent creep behaviors in the long-term deformation. Accordingly, the material properties of the rubber showed hyper-elastic behavior for capturing nonlinearity elasticity in the initial deflection range and a time-related damage function for representing nonlinear displacement–time relationships in the long-term creep range. Therefore, the constitutive equation of rubber materials is denoted as [17]

*W* = *W*_hyper_ + *W*_creep_, (1)

where *W*_hyper_ and *W*_creep_ are the hyper-elastic model and the time-decay creep model, respectively.

In general form, *W*_hyper_ is denoted by,

(2) $$W_{hyper}=W\left(\bar{I}\right)+W\left(J\right),$$

where *W(*$\bar{I}$*)* is the deviatoric part of the strain energy density of the primary material response and *W(J)* is the volumetric part of the strain energy density. For isotropic rubber, *W(*$\bar{I}$*)* depends on strain invariants, ${\bar{I}}_{1}$, ${\bar{I}}_{2}$, and ${\bar{I}}_{3}$. Strain invariants can be expressed in terms of three principle stretch ratios, $\lambda_{1}$, $\lambda_{2}$, and $\lambda_{3}$, and it is noted that, for incompressible rubbers, $\lambda_{3}$ is 1.

(3) $${\bar{I}}_{1}=\lambda_{1}^{2}+\lambda_{2}^{2}+\lambda_{3}^{2}$$

(4) $${\bar{I}}_{2}=\lambda_{1}^{2}\lambda_{2}^{2}+\lambda_{2}^{2}\lambda_{3}^{2}+\lambda_{3}^{2}\lambda_{1}^{2}$$

(5) $${\bar{I}}_{3}=\lambda_{1}^{2}\lambda_{2}^{2}\lambda_{3}^{2}$$

In *W(J)*, *J* is Jacobian of the deformation gradient and it is a measure of the volume change caused by a deformation

(6) $$J=\sqrt{\det(B)}=\det(F),$$

where *F* is the deformation gradient tensor.

(7) $$F=\left\{\begin{array}{ccc} 1+\frac{\partial u_{1}}{\partial x_{1}} & \frac{\partial u_{1}}{\partial x_{2}} & \frac{\partial u_{1}}{\partial x_{3}}\\ \frac{\partial u_{2}}{\partial x_{1}} & 1+\frac{\partial u_{2}}{\partial x_{2}} & \frac{\partial u_{2}}{\partial x_{3}}\\ \frac{\partial u_{3}}{\partial x_{1}} & \frac{\partial u_{3}}{\partial x_{2}} & 1+\frac{\partial u_{3}}{\partial x_{3}} \end{array}\right\},$$

where *u*_1_, *u*_2_, and *u*_3_ are three-dimensional deformation and *x*_1_, *x*_2_, and *x*_3_ are three-dimensional coordinate axis. For stress calculation of hyper-elasticity, the strain energy potential in polynomial series is expressed as

(8) $$W_{hyper}=\sum_{i+j=1}^{N}C_{ij}{\left({\bar{I}}_{1}-3\right)}^{i}{\left({\bar{I}}_{2}-3\right)}^{j}+\sum_{i=1}^{N}\frac{1}{D_{ij}}{\left(J_{el}-1\right)}^{2i},$$

where $C_{ij}$ and $D_{i}$ are temperature-dependent material parameters and $J_{el}$ is elastic volume strain.

Using Equation (8), some typical hyper-elastic models are derived. For example, if *N* = 0, the polynomial formulation represents the Neo–Hookean model, which is written as

(9) $$W_{hyper-NH}=C_{10}\left({\bar{I}}_{1}-3\right).$$

If N = 1, the Mooney–Rivlin hyper-elastic model is obtained as

(10) $$W_{hyper-MN}=C_{10}\left({\bar{I}}_{1}-3\right)+C_{01}\left({\bar{I}}_{2}-3\right)+\frac{1}{D_{1}}{\left(J_{el}-1\right)}^{2}.$$

Also, using the modified Equation (10), the Yeoh hyper-elastic model can be obtained:

(11) $$W_{hyper-Yeoh}=C_{10}\left({\bar{I}}_{1}-3\right)+C_{20}{\left({\bar{I}}_{1}-3\right)}^{2}+C_{30}{\left({\bar{I}}_{2}-3\right)}^{3}.$$

The hyper-elastic constitutive equation of rubber can be also expressed in high-order polynomial form. For easy implementation and reasonable accuracy, the strain energy potential in terms of Mooney–Rivlin was adopted in the present work. Hyper-elastic material parameters (*C_01_* and *C_10_*) at different temperatures were evaluated by the quasi-static experimental results. The detailed identification is: Using the experimental force-deformation curve as shown in Figure 4, nominal strain (change in length per unit of original length) and nominal stress (force per unit of original cross-sectional area) are derived. Given experimental nominal stress–strain results, the parameters of the hyper-elastic model are determined by utilizing the least squares fitting algorithm [23]. The identified objective is to minimize the relative error, *E_e_*.

(12) $$E_{e}={\sum_{i=1}^{n}\left(1-\frac{T_{i}^{th}}{T_{i}^{test}}\right)}^{2},$$

where $T_{i}^{test}$ is the stress from the test results and $T_{i}^{th}$ is the nominal stress.

Hyper-elastic models as shown in Equation (2–11) are suitable to depict loading portion in a mechanical process; however, the unloading process cannot be predicted. Using a rebound energy approach [24], a modified hyper-elastic model for describing the complete loading–unloading process is developed:

(13) $$W_{hyper}=\left[1-\left(1-\theta_{0}\right)\beta \right]W\left(\bar{I}\right)+W\left(J\right),$$

where $\theta_{0}$ is rebound resilience parameter of rubbers and β is a state variable. In the loading process, β is 0. In the unloading process, at the beginning of unloading, β is 0 and at the end of unloading, β is 1. In the present work, $\theta_{0}$ is 0.55.

As shown in Equation (1), a time-decay function with a damage concept, *W*_creep_, is incorporated into a constitutive equation of rubbers, which describes the nonlinear creep behaviors considering material constitutive structure change and the elapsed time. The creep damage model should be assumed as the creep effect from the deviatoric strain invariants during loading, ${\bar{I}}_{1}$ and ${\bar{I}}_{2}$, and also the elapsed loading time, *t*. For characterizing nonlinear creep responses, a phenomenological mathematic model of *W*_creep_ is developed. In this work, three kinds of widely nonlinear decay functions are utilized for developing creep damage constitutive models and compared in terms of accuracy, which is in the form of power-law functions, logarithmic functions, and exponential functions. As shown in experimental results, creep parameters should be varied with the temperature.

(1) Power-law creep constitutive model is expressed:

(14) $$W_{creep}=k_{1}\left(T\right)t^{r_{1}\left(T\right)}({\bar{I}}_{1}+{\bar{I}}_{2}).$$

(2) Logarithmic-based creep constitutive model is shown:

(15) $$W_{creep}=\left[k_{2}\left(T\right)\log_{r_{2}\left(T\right)}t\right]({\bar{I}}_{1}+{\bar{I}}_{2}).$$

(3) Exponential-based creep constitutive model is expressed:

(16) $$W_{creep}=k_{3}(T)e^{r_{3}\left(T\right)t}({\bar{I}}_{1}+{\bar{I}}_{2}),$$

where *k_i_* and *r_i_* (*i* = 1, 2, 3) are creep parameters. A trial and error procedure was arranged so that the best adjustment of creep responses could be achieved between numerical and experimental results, and hence creep parameters were identified.

### 3.2. Implementation of Finite Element Method

**Figure 7:**
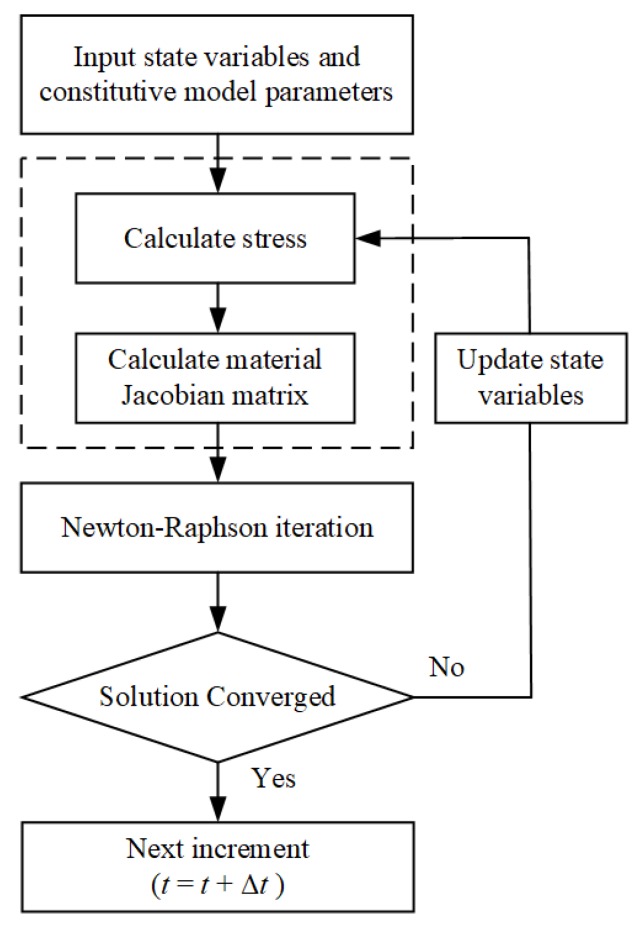
Flow chart of UHYPER implementation in ABAQUS.

The numerical analysis was conducted by the finite element method using Abaqus software. In material libraries, only standard hyper-elastic models are available, such as Neo–Hooke, Mooney–Rivlin, Ogden, Yeoh, polynomial-term, van der Waals, and Arruda–Boyce. In the present work, the proposed model is not a standard model and hence needs to be incorporated via user subroutine. As the UHYPER user subroutine defines the increments of hyper-elastic strain and time-dependent inelastic strain, which is the function of the solution-dependent variables, e.g., deviatoric stress, loading, time-step increment, and temperature [25]. Abaqus provides both explicit and implicit time integration of creep and the choice of the time integration scheme depends on the procedure type, the procedure definition, and a geometric non-linearity [1]. The flow chart of implementation of constitutive model by UHYPER is illustrated in Figure 7.

**Figure 8:**
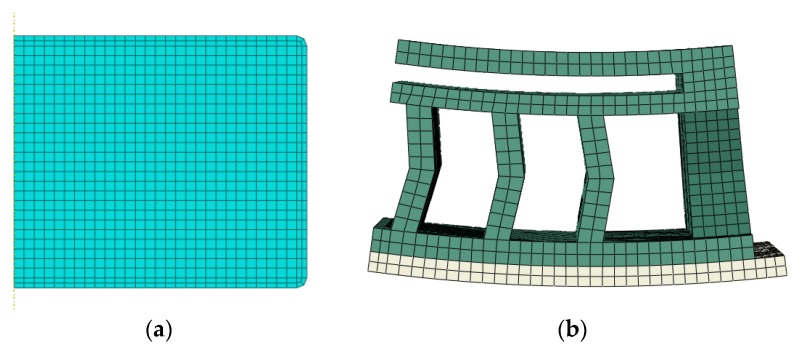
Numerical model of rubber samples. (**a**) Rubber materials, (**b**) rubber device.

The finite element model of rubber with mesh and boundary conditions according to the experimental arrangement was established. Due to the symmetry of the rubber sample’s geometry and the loading condition, an axial symmetric model was established. CAX4HT, which is a 4-node thermally-time coupled plan element with 3 degrees of freedom, was utilized to mesh the rubber isolator. In the finite element model, the total number of nodes and elements were 1907 and 838, respectively. For the rubber vibration system, its finite element model was developed by C3D8HT element with the total number of nodes and element being 63949 and 24153. The symmetric boundary condition was applied in the symmetric plan and the bottom of the rubber was constrained. The loading force was applied on a rigid body along the vertical direction and the degrees of freedom of the rigid body and the rubber system were coupled. The numerical models are shown in Figure 8. In addition, the rigid body and rubber materials were applied using surface-to-surface contact conditions to prevent interpenetration and the friction coefficient value was 0.2.

## 4. Results and Discussions

### 4.1. Quasi-Static Analysis

**Figure 9:**
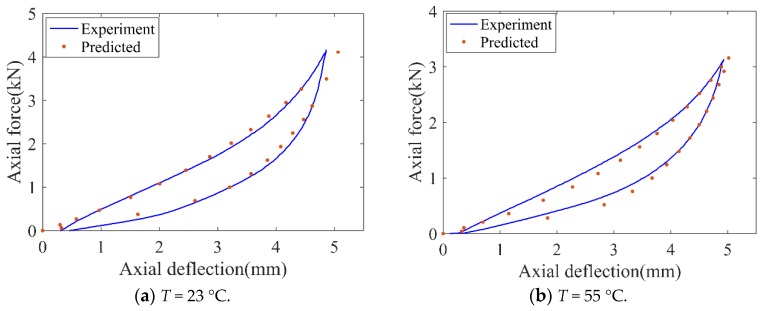
Comparison of load-deflection curves between the simulation and the experiment.

Since the proposed constitutive model in this study is based on strain energy potential, the validation of the hyper-elastic model is fundamental for creep analysis. Load-deflection histories of the simulation and experiment resulting in quasi-static compression are compared in Figure 9.

As shown in Figure 9, the curves of the numerical and the experimental results are consistent, which implies the model could accurately predict the deformation process in the static loading–unloading compression.

**Figure 10:**
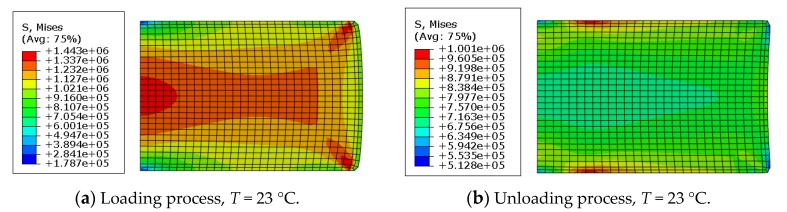

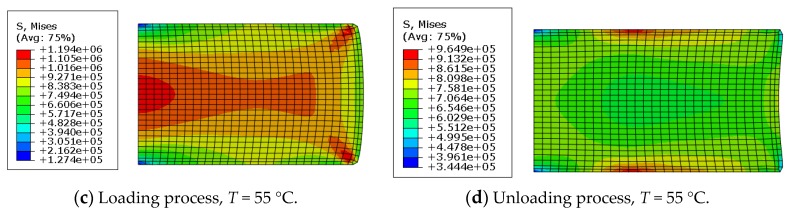
Stress distribution profile at strain of 16%.

Figure 10 shows the stress profile of the rubber in the identical deformation (ɛ = 0.16) at two temperatures.

In Figure 10, it is shown that the stress distribution presents symmetrically, which is due to the loading and boundary conditions. In the loading process, as shown in Figure 10a,c, rubber showed swelling in the radial boundary under compression and in the unloading process in Figure 10b,d, the rubber was elongated under rebound deformation. The maximum Mises stress points at different temperatures were mainly located on the contact edges. Such stress concentration was induced by the non-flat surface of the rubber in the contact area, which was the result of uncontrolled slippage at the rubber–rigid interface [18]. Additionally, at the strain of 16%, the maximum stress values in the loading process are 1.44 MPa and 1.19 MPa at 23 °C and 55 °C, respectively, which can be explained by the stiffness analysis as compared in Figure 4. Compared with Figure 10a,b at 23 °C under the same strain, the maximum stress in loading (1.44 MPa) is larger than that (1.00 MPa) in unloading; similar behaviors are also shown at 55 °C, hence the numerical model could evaluate the Mullins effect.

### 4.2. Creep Analysis

The creep numerical simulation was performed in accordance with the experimental test, in which three levels of loading of 1.5 kN, 2.0 kN, and 2.5 kN at two temperatures (23 °C and 55 °C) were held for 48 h, respectively.

**Figure 11:**
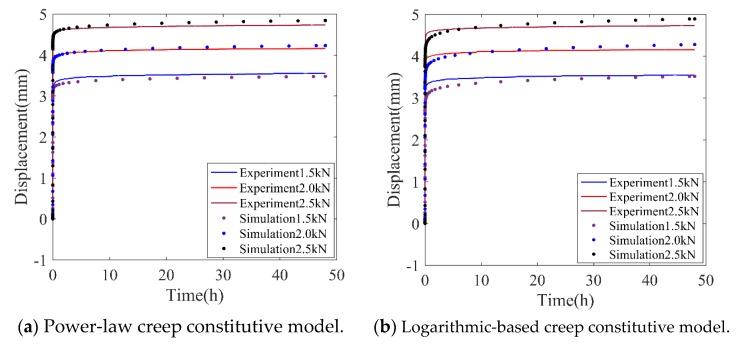

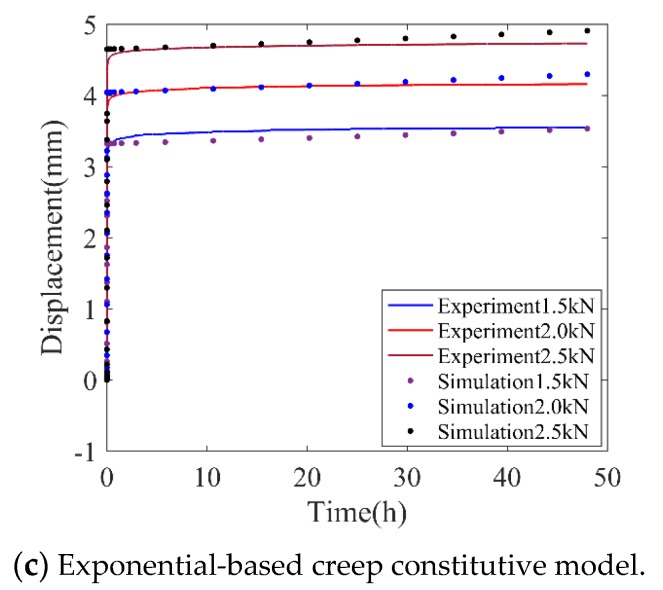
Comparison of creep deformation between numerical and experiment results (*T* = 23 °C).

To validate the reliability of the numerical simulation and compare the accuracy of the three creep damage functions, the creep deformations obtained from the simulation are presented and compared with experimental results in Figure 11.

In Figure 11, it is seen that the proposed creep damage functions generally could predict the time-dependent increasing deformation in creep tests under different levels of loadings and temperatures. Compared with power-law and the logarithmic-based creep constitutive model, the exponential-based creep constitutive model showed steep deformation in the initial creep deformation, which indicates that this model is not suitable for predicting the primary creep stage. As for the logarithmic-based model, the long-term behavior is reasonably evaluated but it shows a relatively large error in the amplitude of initial creep deformation.

For clarification, the error analysis of these creep constitutive models is presented in Table 1. Several error indexes are selected, such as the squared correlation coefficient (SCC), the mean absolute percentage error (MAPE) and the mean square error (MSE). The expressions of error indexes are provided in Table 2.

As shown in Table 1, the SCC of these three models were all larger than 0.99, which verifies the validation of these adopted time-dependent nonlinear creep models. For the other two indexes, compared with logarithmic-based and exponential-based models, the average value of MAPE of the power-law creep model decreased by 46.8% and 55.4%, respectively. In the case of 2.0 kN applied loading, the MSE of the power-law creep model decreased by 58.5% and 85.9% respectively than that of logarithmic-based and exponential-based models. Therefore, the power-law creep constitutive model was chosen for the subsequent creep analysis because of the high accuracy.

**Figure 12:**
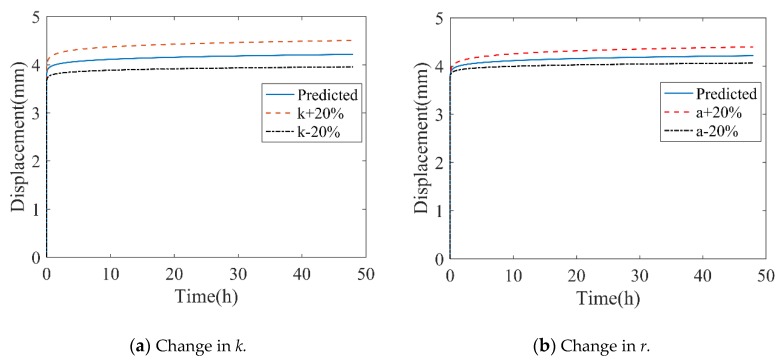
Effects of varying parameters in creep deformation responses.

To further study the effect of creep parameters on responses in the power-law creep model, sensitivity analysis was conducted. The detailed process of parameter sensitivity analysis is as follows: (1) A set of creep parameters as the reference values was selected. In the present work, the reference parameters were adopted in testing conditions of 2.0 kN loading at 23 °C, denoted as *k*_0_ and *r*_0_. (2) One parameter’s value was varied and another was unchanged; the changed proportion range of each parameter was approximately –20% to +20%. The effects of varying parameters are shown in Figure 12.

As shown in Figure 12, when *k* was equal to 0.8 *k*_0_ and 1.2 *k*_0_, the maximum deformations in creep were 0.93 *δ*_0_ and 1.07 *δ*_0_, respectively, in which *δ*_0_ was the maximum deformation of *k*_0_ and *r*_0_. For varying *r*, the difference in maximum deformations was 0.96*δ*_0_ and 1.04*δ*_0_ for +20% *r*_0_ and −20% *r*_0_, respectively. In the phenomenal aspect, *k* controlled the amplitude of creep deformation, which describes the strength of the creep damage, and *r* determines the inclination degree of creep deformation.

**Figure 13:**
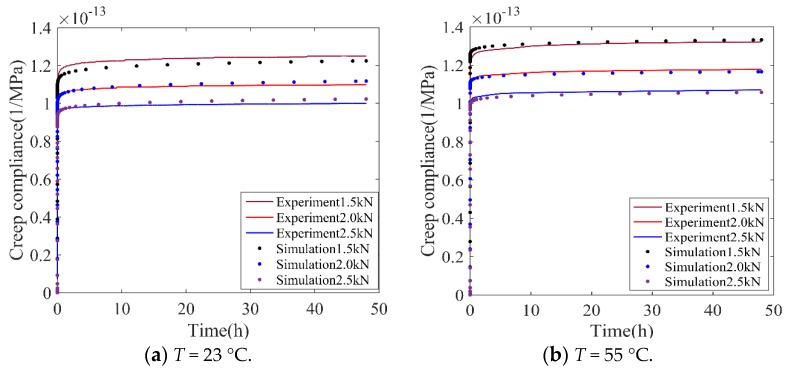
Comparison of numerical and experimental creep compliance.

Creep compliance, *J*(*t*), is a representative index for evaluating creep performances. Using the power-law creep model, *J*(*t*) under different levels of loading and temperature is presented in Figure 13.

As shown in Figure 13, it was concluded that when the loading levels increased, the creep compliance reduced. This phenomenon is presented at different temperatures; additionally, at the same loading level, the increment of temperature leads to the enhancement of creep compliance.

**Figure 14:**
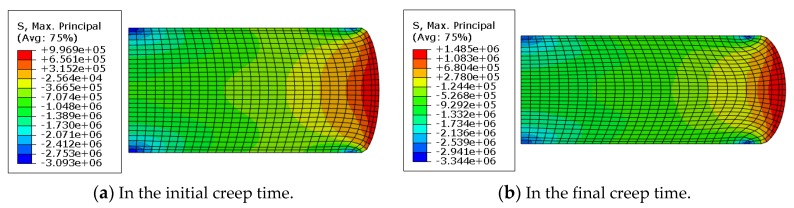
Maximum principle stress profiles (*T* = 23 °C; *F* = 2.5 kN).

During creep, the stress distribution of rubber changes with time. However, these results cannot be observed by experimental testing. In the present work, the maximum principle stress profiles of rubbers are studied by finite element simulation, as shown in Figure 14.

**Figure 15:**
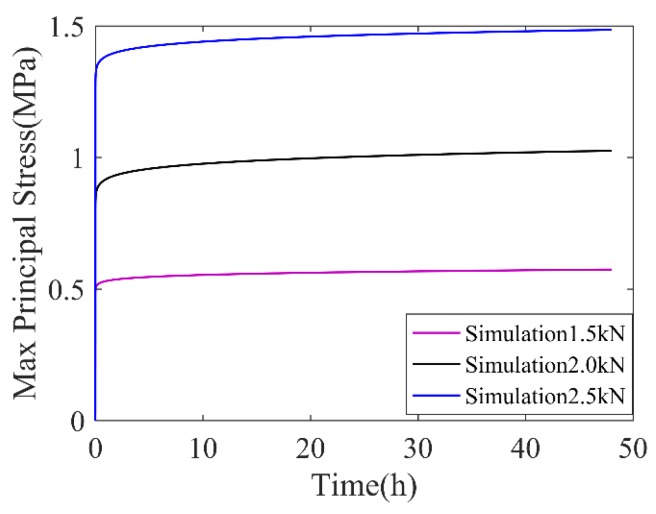
Time variation of maximum principle stress of reference point (*T* = 23 °C).

In Figure 14, it is shown that the tensile stress distributes around the free surfaces and the maximum tensile principle stress is observed at the center of the free surface, while the maximum compressive stress occurs at the contact edge. Compared with Figure 14a,b, the maximum tensile stress was 0.99 MPa in the initial creep and 1.48 MPa in the final creep, which indicates that the maximum principle stress was enhanced during creep process. For detailed comparison, the time-dependent maximum principle stress of a reference point where the maximum tensile principle stress was located is plotted in Figure 15.

In Figure 15, it is clearly seen that, at a fixed loading level, the maximum principle stress of the reference point increased over time. This phenomenon is called “stress hardening”, which can be explained by the engineering principle, in that the extra geometric deformation during creep adds to the mechanical deformation [17]. It is also shown that, with the increase in loading levels, the maximum principle stress and its hardening degree (the slope of the time varied maximum principle stress) increased.

To study the effect of temperature and loading levels on the stress hardening effect, the maximum tensile principle stresses under different conditions are compared in Table 3. The hardening degree, λ, is calculated as

(17) $$\lambda (\%)=\left(\frac{\sigma_{\max-f}-\sigma_{\max-i}}{\sigma_{\max-i}}\right)\times 100\%,$$

where $\sigma_{\max-f}$ and $\sigma_{\max-i}$ are the maximum tensile principle stresses in the final and initial creep times, respectively.

As shown in Table 3, at room and high temperatures, $\sigma_{\max-i}$ and $\sigma_{\max-f}$ increased with increasing loading level. It was also seen that λ at room temperature was in the range of 40–50%, while at high temperatures, λ was less than 10%. Hence, a slower development of maximum principle stress increase occurs at high temperature.

**Figure 16:**
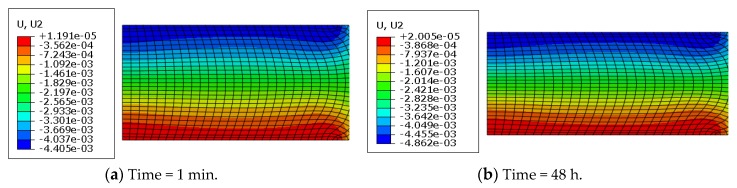
Axial creep deformation profiles at different times (*T* = 23 °C; *F* = 1.5 kN).

Additionally, the axial creep deformation profiles at different times are shown in Figure 16.

As shown in Figure 16, the axial deformation profiles presented a layer phenomenon: At the bottom, it was the minimum (approximately zero) due to the boundary condition, at the top, it was the maximum because of the loading condition, and in other areas it gradually varied. This layer characteristic stayed unchanged over different creep times. Additionally, the maximum creep deformation increased over time and bulging of the free surfaces enhanced with increasing time.

To evaluate the creep degree, the creep deformation percentage, *Creep* (%), under different loading levels and temperatures, is compared in Table 4.

(18) $$Creep(\%)=\left(\frac{D_{t}-D_{0}}{D_{e}-D_{0}}\right)\times 100\%,$$

where *D_t_* is the creep deformation after *t*, *D_e_* is the final creep deformation, and *D*_0_ is the creep deformation at the end of applied loading.

As shown in Table 4, at a fixed loading level, the creep deformation percentage in room temperature was higher than that in high temperature; this temperature-related phenomenon is increasingly obvious with increasing loading. For example, at the time interval of 1 min, the difference in creep deformation percentage between 23 °C and 55 °C, δ, is 0.39% at a loading of 1.5 kN and 4.9 % at a loading of 2.5 kN, a 12.6 times increase. In addition, δ changes in different creep stages. For instance, when the applied loading was 2.5 kN, δ was 17.28% at 30 min and 4.79% at 24 h. Hence, at a fixed applied loading, δ in initial creep was greater than that in stable creep, which indicates that high temperature leads to a faster development of creep deformation in stable creep.

Relative creep rate (*RC*) is another significant index for depicting creep behaviors, which is defined as [16]

(19) $$RC=\left(\frac{D_{t}-D_{0}}{H}\right),$$

where *D_t_* is the creep deformation after t and *H* is the original thickness.

**Figure 17:**
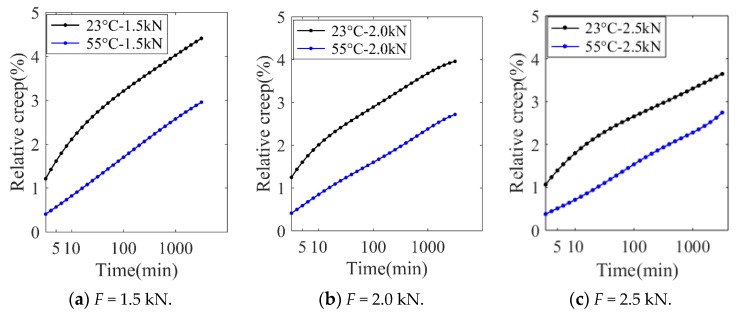
Creep rate for rubbers under different conditions.

Figure 17 shows the relative creep rate versus time plot.

As shown in Figure 17, under different loading levels and temperatures the relative creep rate is approximately proportional to the logarithm of time in the stable creep stage, which characteristic of physical creep [26]. Physical creep is due to the viscoelasticity of rubber materials and the slippages in cross links of rubbers molecules under loading. In general, physical creep is primarily dominated in short-time creep tests (less than 10^3^ min). It was also seen that, at room temperature, *RC* at loading levels of 1.5 kN, 2.0 kN, and 2.5 kN were 4.37%, 3.95%, and 3.61%, respectively, while in high temperatures, *RC* at the final creep time at loading levels of 1.5 kN, 2.0 kN, and 2.5 kN were 2.90%, 2.71%, and 2.69%, respectively. These results demonstrate that the high temperature mode shows more creep resistance than room temperature at a fixed loading. This can be explained by a slower increase of maximum principle stress due to “stress hardening” and a more uniform stress distribution in the case of high temperature, as shown in Table 3.

**Figure 18:**
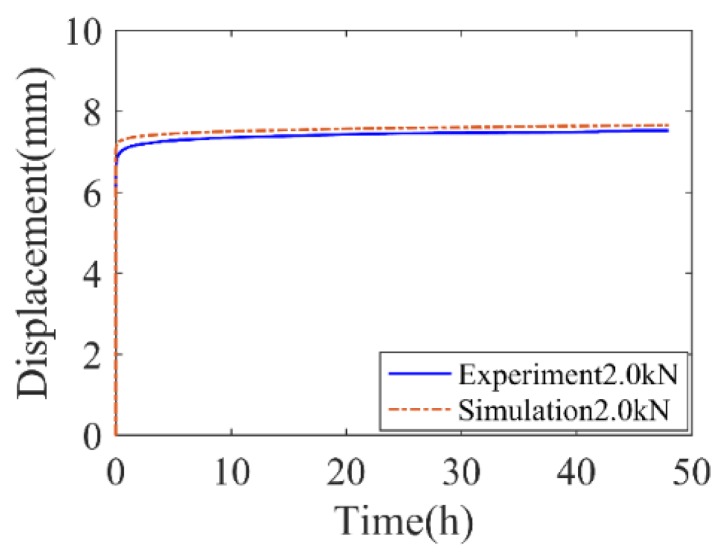
Comparison of numerical and experimental creep deformation of the rubber system at room temperature.

To further evaluate the engineering potential of the proposed constitutive model, the creep behavior of the rubber vibration system was predicted and compared with experimental results, as shown in Figure 18. It is seen that the proposed model in which the materials’ parameters are identified by rubber-material testing could depict the different creep stages of the rubber system and the numerical solutions match the experimental results.

**Figure 19:**
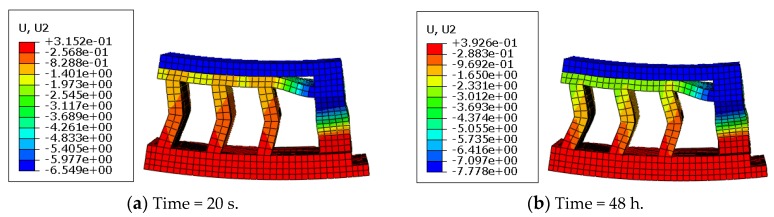
Creep profiles of the rubber system at different times (*T* = 23 °C; *F* = 2.0 kN).

The axial creep deformation profiles at different times are shown in Figure 19.

In Figure 19, it is seen that the deformation distribution at different creep times was similar; however, the maximum creep deformation increased with time elapsing. It is noticed that at the initial creep time (20 s), self-contact had occurred inside the top of the rubber system, which lasted during the creep deformation. Different from rubber materials, the axial creep deformation presented an irregular layer phenomenon due to the structural geometry effect.

**Figure 20:**
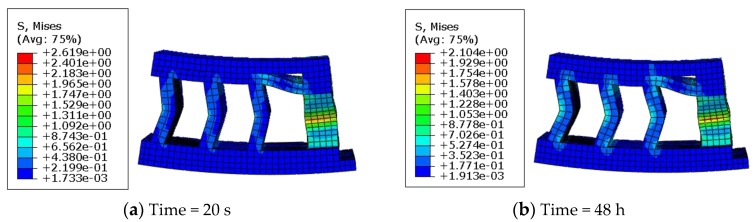
Mises stress distribution of the rubber system at different creep times (*T* = 23 °C; *F* = 2.0 kN).

During creep, the Mises stress distribution of the rubber system at different creep times is shown in Figure 20.

As seen in Figure 20, the patterns of the two stress profiles look similar; the maximum Mises stress was located in the region where the stiffness is weakest (e.g., the central region). Using the analysis of the rubber system mentioned above, the proposed model and its numerical approach could provide a good prediction for creep evaluation of rubber-based engineering cases.

## 5. Time–Temperature Equivalent Analysis

It is evident that creep responses measured by long-term loading creep tests are expensive and time-consuming. To reduce the experimental cost, time–temperature equivalent analysis should be conducted. Some accelerated methods have been developed to predict long-term creep performances of rubber materials based on short-time experiments [27,28]. The principle of these accelerated methods lies in the fact that, in the creep test, the effect of longer time is similar to the effect of higher temperature [29]. Among them, the well-known equation for describing the temperature–time equivalent principle is Williams–Landel–Ferry (WLF) equation [30]; its time-dependent shift factor, $\alpha_{T}$, is expressed as

(20) $$Log(\alpha_{T})=\frac{-C_{1}\left(T-T_{r}\right)}{C_{2}+(T-T_{r})},$$

where *C*_1_ and *C*_2_ are two constants which are related to the reference temperature *T_r_* and the type of rubber materials. According to ISO4664-1, when the glass transition temperature (*T_g_*) is regarded as the reference temperature (*T_r_*), then *C*_1_ and *C*_2_ are 17.44 K and 51.6 K, respectively [31]. Then, the time–temperature equivalent shift factor calculated in the term of glass transformation temperature is

(21) $$Log(\alpha_{T})=\frac{-17.44\left(T-T_{g}\right)}{51.6+(T-T_{g})}.$$

One of the key issues of applying the WLF equation is the determination of the glass transition temperature of rubber materials. In the present work, the glass transformation temperature was tested utilizing the dynamic differential scanning calorimetry (DSC) method [31]. Measurements were conducted on a Mettler–Toledo DSC instrument (Type: QJ-X03) in a fluid nitrogen atmosphere. Rubber samples were prepared weighing 5.80 mg of the compound in aluminum crucibles. The weight was measured by the Mettler–Toledo scale (type: XP105) with a resolution of 0.01mg. Before testing, the rubbers were heated from −100 °C to 100 °C for erasing in-balance thermal effects, then this was repeated using constant heating and cooling rates of 10 °C/min. During DSC measurement, liquid nitrogen was released at 10 mL/min.

**Figure 21:**
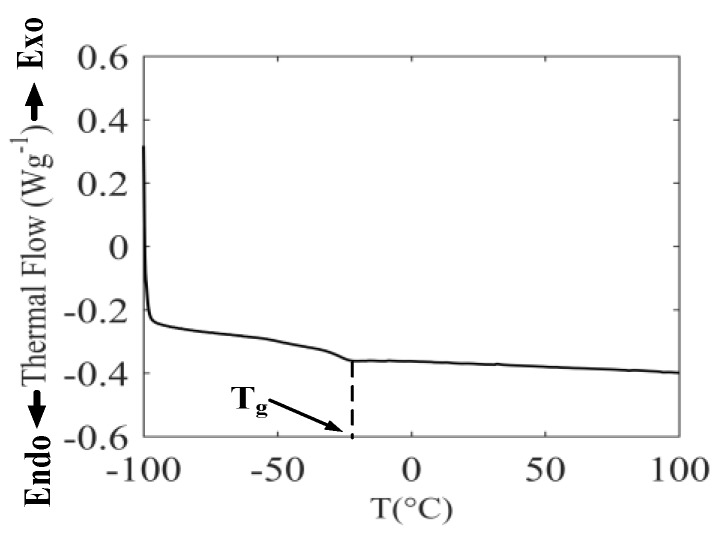
Determination of *T_g_* by differential scanning calorimetry (DCS) method.

The thermal flow curve of rubber samples is shown in Figure 21. The inflection point of this figure represents the glass transition temperature of rubber samples, hence the glass transition temperature was –25.70 °C as shown by data processing. Additionally, according to GB/T 29611-2013, titled as “Determination of the rubber’s glass transition temperature by differential scanning calorimetry (DSC) method”, the labels of exo and endo are added in Figure 21.

**Figure 22:**
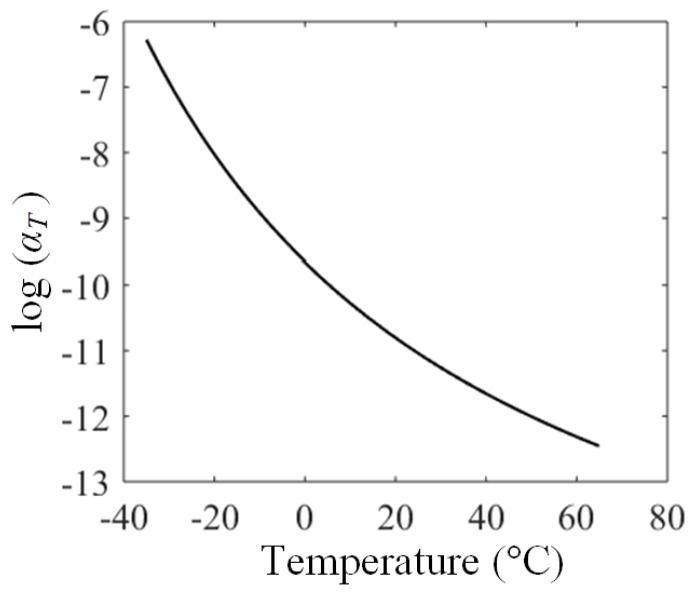
Temperature dependence of instantaneous modulus of rubber materials.

By integrating glass transition temperature into Equation (21), the master curve of the equivalent shift factor, $\alpha_{T}$, is illustrated in Figure 22.

Based on the equivalent shift factor master curve, the creep compliance, *J*(*T*_1_) at temperature *T*_1_, can be converted to the creep compliance, *J*(*T*_2_) at temperature *T*_2_ [31], which is expressed as

(22) $$J\left(T_{1}\right)=J\left[\log\left(\frac{\alpha_{T_{1}}}{\alpha_{T_{2}}}\right)\cdot T_{2}\right].$$

## 6. Conclusions

This paper provides a hyper-elastic creep constitutive model to evaluate creep characteristics of rubber materials under different conditions. The numerical implementation of the proposed phenomenological model is presented and validated. The time-dependent, loading-related, and temperature-induced creep behaviors of rubber materials are studied. The proposed method is further utilized to predict creep performances of a rubber vibration system for validating its engineering potential. A time-temperature equivalent analysis by WLF equation in glass transition temperature is also introduced. By comparing numerical and experimental results, the proposed creep models could depict nonlinear creep behaviors of rubber materials and rubber vibration systems, which provides an option for rubber system design and its creep prediction.

## Figures and Tables

**Table 1:** Error analysis of different creep damage functions (*T* = 23 °C).

Creep Models	Loading	Error Indexes		
		SCC	MAPE	MSE
Power-low function	1.5 kN	0.9991	2.9821	0.0113
	2.0 kN	0.9996	1.7582	0.0093
	2.5 kN	0.9998	1.6917	0.0087
Logarithmic function	1.5 kN	0.9995	4.9595	0.0303
	2.0 kN	0.9990	3.4315	0.0224
	2.5 kN	0.9988	3.7001	0.0337
Exponential function	1.5 kN	0.9959	5.3389	0.0497
	2.0 kN	0.9976	4.7459	0.0661
	2.5 kN	0.9986	4.3447	0.0633

**Table 2:** Expression of error evaluation indexes.

Error Index	Formula	Analysis
SCC	${\left\{\frac{\sum_{i=1}^{n}\left[F_{sim}\left(i\right)\cdot F_{\exp}\left(i\right)\right]}{\sqrt{\sum_{i=1}^{n}F_{sim}{\left(i\right)}^{2}}\cdot \sqrt{\sum_{i=1}^{n}F_{\exp}{\left(i\right)}^{2}}}\right\}}^{2}$	Larger is better
MAPE	$\left(\frac{100}{n}\right)\cdot \sum_{i=1}^{n}\left|\frac{F_{sim}\left(i\right)-F_{\exp}\left(i\right)}{F_{\exp}\left(i\right)}\right|$	Smaller is better
MSE	$\frac{1}{n}\cdot {\sum_{i=1}^{n}\left|F_{sim}\left(i\right)-F_{\exp}\left(i\right)\right|}^{2}$	Smaller is better

**Table 3:** Maximum tensile principle stress under different conditions.

Temperature	Loading	$\sigma_{\max-i}\;(\mathrm{MPa})$	$\sigma_{\max-f}\;(\mathrm{MPa})$	λ (%)
23 °C	1.5 kN	0.0413	0.0598	44.95%
	2.0 kN	0.0669	1.0025	53.21%
	2.5 kN	0.0997	1.0485	48.95%
55 °C	1.5 kN	0.0403	0.0439	8.93%
	2.0 kN	0.0645	0.0691	7.13%
	2.5 kN	0.0913	0.0923	1.10%

**Table 4:** *Creep* (%) of rubber materials during the creep test at different times.

Loading		1.5 kN		2.0 kN		2.5 kN	
Temperature		23 °C	55 °C	23 °C	55 °C	23 °C	55 °C
Time	1 min	27.17%	26.78%	31.90%	29.50%	30.71%	25.81%
	30 min	72.50%	58.88%	74.31%	60.50%	74.28%	57.00%
	1 h	77.50%	66.70%	78.62%	68.14%	78.50%	61.55%
	6 h	89.33%	83.27%	88.97%	80.65%	88.48%	81.86%
	12 h	92.67%	89.07%	93.97%	89.23%	92.32%	85.94%
	24 h	96.00%	95.42%	97.24%	94.69%	96.16%	91.37%
	48 h	100.00%	100.00%	100.00%	100.00%	100.00%	100.00%
